# Sensitivity of Small RNA-Based Detection of Plant Viruses

**DOI:** 10.3389/fmicb.2018.00939

**Published:** 2018-05-14

**Authors:** Johanna Santala, Jari P. T. Valkonen

**Affiliations:** ^1^Finnish Food Safety Authority Evira, Helsinki, Finland; ^2^Department of Agricultural Sciences, University of Helsinki, Helsinki, Finland

**Keywords:** plant virus, diagnostics, small RNA, siRNA, detection threshold, VirusDetect, qPCR

## Abstract

Plants recognize unrelated viruses by the antiviral defense system called RNA interference (RNAi). RNAi processes double-stranded viral RNA into small RNAs (sRNAs) of 21–24 nucleotides, the reassembly of which into longer strands *in silico* allows virus identification by comparison with the sequences available in databases. The aim of this study was to compare the virus detection sensitivity of sRNA-based virus diagnosis with the established virus species-specific polymerase chain reaction (PCR) approach. Viruses propagated in tobacco plants included three engineered, infectious clones of *Potato virus A* (PVA), each carrying a different marker gene, and an infectious clone of *Potato virus Y* (PVY). Total RNA (containing sRNA) was isolated and subjected to reverse-transcription real-time PCR (RT-RT-PCR) and sRNA deep-sequencing at different concentrations. RNA extracted from various crop plants was included in the reactions to normalize RNA concentrations. Targeted detection of selected viruses showed a similar threshold for the sRNA and reverse-transcription quantitative PCR (RT-qPCR) analyses. The detection limit for PVY and PVA by RT-qPCR in this study was 3 and 1.5 fg of viral RNA, respectively, in 50 ng of total RNA per PCR reaction. When knowledge was available about the viruses likely present in the samples, sRNA-based virus detection was 10 times more sensitive than RT-RT-PCR. The advantage of sRNA analysis is the detection of all tested viruses without the need for virus-specific primers or probes.

## Introduction

The large number and high genetic variability of plant viruses make their detection cumbersome. Virus-specific antibodies or probes are available mainly for prevalent, economically harmful plant viruses, whereas the absence of specific diagnostic tools for the great majority of plant viruses hampers broader surveys of viruses (Zaitlin and Palukaitis, 2000; Jones et al., 2017). Plants themselves do, however, recognize virus infection via an antiviral defense system called RNA interference (RNAi), which targets and processes double-stranded RNA into 21- to 24-nucleotide (nt) small RNAs (sRNAs) (Ding and Voinnet, 2007). sRNAs can be extracted from plant tissues and sequenced, and the sequences can then be reassembled into partial or full viral genomes *in silico*, which allows identification by comparison with viral sequences available in databases. This method, published by Kreuze et al. (2009), has been widely adopted and has been used for surveys of viruses in cultivated (Kashif et al., 2012; Moon and Park, 2016; Massart et al., 2017) and wild plants (Bi et al., 2012). It allows the detection of different, unrelated viruses simultaneously in a single assay, without need for antibodies, probes, etc.

The real-time polymerase chain reaction (RT-PCR) method is a well-established, sensitive and specific tool for virus detection and was used for many years before the sRNA-based approach was introduced (Rowhani et al., 1995; Roberts et al., 2000; Boonham et al., 2008; Gutiérrez-Aguirre et al., 2009; Mirmajlessi et al., 2015). RT-PCR-based methods will likely remain as basic tools for plant inspection, e.g., targeted testing of viruses, but detection based on RNA sequencing is gaining popularity for screening a wide range of viruses simultaneously. As interest in applying sRNA-based virus detection in official plant inspection is increasing, the aim of this study was to estimate whether similar detection thresholds can be reached with the sRNA-based and RT-PCR-based methods.

## Materials and Methods

### Virus Propagation

Infectious clones of *Potato virus A* (PVA) engineered to express red fluorescent protein (RFP) (PVA-ynHC/6K2rfp) (Haikonen et al., 2013), green fluorescent protein (PVA-GFP) (Rajamäki et al., 2005) or β-glucuronidase (PVA-GUS) (Kelloniemi et al., 2008) and an infectious clone of *Potato virus Y* [PVY-N605(1048)] (Bukovinszki et al., 2007) were used to inoculate 5-week-old tobacco plants (*Nicotiana tabacum* L. cv. Samsun nn) by particle bombardment using a HandyGun (Sikorskaite et al., 2010). Plants were placed in a growth chamber (16-h photoperiod; relative humidity, 70%; light intensity, 200 μE m^-2^s^-1^; temperature, 18 and 22°C during the night and day, respectively) and were given 0.3 g fertilizer (16:9:22 = N/P/K; Yara, Espoo, Finland) per liter of water when watered.

### RNA Extraction, Quantification, and cDNA Synthesis

The uppermost fully expanded leaves were sampled from the tobacco plants. Total RNA was extracted from the leaves using Trizol reagent generated in-house (Caldo et al., 2004). In addition, pure viral RNA was isolated with this method from virus particles that had been isolated from tobacco leaf sap, as described (Valkonen et al., 1991). The RNA concentration was determined using a Nanodrop (Thermo Fischer Scientific, Waltham, MA, United States). 1 μg of leaf RNA (per sample) was treated with RQ1 RNase-free DNase (Promega, Madison, WI, United States) according to the manufacturer’s instructions and used for cDNA synthesis. cDNA synthesis was also carried out on 1000 and 250 ng of RNA extracted from purified PVY and PVA particles, respectively. cDNA was synthesized with *Moloney murine leukemia virus* (M-MLV) reverse transcriptase (Promega) using random hexamers, according to the manufacturer’s instructions.

Total RNA was also isolated from leaves of cultivated strawberries [Fragaria × ananassa (Weston) Duchesne ex Rozier (pro sp.)], raspberries (*Rubus idaeus* L.) and *Ribes* species obtained from the genetic resource collection maintained by the Natural Resources Institute of Finland (LUKE). A protocol combining the cetyl trimethylammonium bromide (CTAB) method and acidic phenol–chloroform extraction was used for RNA purification (Kurokura et al., 2006). Purity and concentration of RNA samples were determined with the Nanodrop. RNA was stored at -80°C.

### RT-PCR and Deep Sequencing

*Potato virus Y* and PVA RNA was quantified by real-time quantitative PCR (qPCR) using cDNA generated from total RNA extracted from systemically infected tobacco leaves. Primer3 software^1^ was used to design specific forward (F) and reverse (R) primers for RT-qPCR amplification of a 127-nt product from the coat protein (CP) encoding region of PVY (F-primer PVY-CP-QPCR-F1, 5′-ACACCAGTGAGGGCTAGRGA-3′; R-primer PVY-CP-QPCR-R, 5′-GTGGTGTGCCTCTCTGTGTT-3′) and a 138-nt product from the CP-encoding region of PVA (F-primer PVA-CP-QPCR-F, 5′-TCGCAGAGGCGTACATTGAG-3′; R-primer PVA-CP-QPCR-R, 5′-CTGATCGGAGTGGTTGCAGT-3′).

Quantitative PCR was carried out using LightCycler 480 SYBR Green I Master Mix (Roche, Basel, Switzerland) and a LightCycler 480 Instrument (Roche) in a 384-well microtiter plate format. Each well in the plate contained 1× SYBR Green I Master Mix with 0.45 pmol of forward and reverse primers and 5 μl (50 ng) of cDNA, in a total volume of 15 μl. The polymerase chain reaction (PCR) program consisted of a pre-incubation step at 95°C for 5 min, followed by 45 cycles of 10 s at 95°C, 10 s at 60°C, and 10 s at 72°C. Production of a single product only by each primer pair was evidenced by melting curve analysis at the end of the program.

For qPCR, the PVY cDNA synthesized from RNA extracted from PVY particles was diluted to 50, 5.0, 0.5, 0.05, or 0.005 ng of cDNA in the reaction. Similarly, 12.5, 1.25, 0.125, 0.0125, or 0.00125 ng of PVA cDNA was included in the qPCR reactions. Cq-values were used to draw a standard curve that was used together with the Cq-values obtained from the PVY- or PVA-infected tobacco leaves to estimate the concentration of viral RNA in tobacco leaves.

For sRNA deep sequencing, different amounts of viral RNA (PVY, PVA-RFP, PVA-GFP, or PVA-GUS) were added to the four RNA pools, each of which contained a total RNA mixture from the leaves of strawberry, raspberry, and *Ribes* spp. These pools contained 3 μg total RNA in a volume of 15 μl. We thus analyzed four different concentrations of PVY and 12 different concentrations of PVA (1.0–0.000001 ng in 3 μg) (**Table 1**).

**Table 1:** Composition of the RNA pools (3 μg) subjected to sRNA deep sequencing.

Pool	Amount of viral RNA (ng)			
	PVY	PVA-GFP	PVA-RFP	PVA-GUS
1	1.0	0.00002	0.01	1.0
2	0.01	0.0002	0.2	0.00001
3	0.0001	0.1	0.000001	0.002
4	0.000001	0.000002	0.0001	0.02


RNA samples sequenced in the laboratory of Fasteris SA (Plan-les-Ouates, Switzerland) were analyzed for RNA concentration with the Qubit RNA assay (Invitrogen) and for integrity with the Agilent Bioanalyzer 2100 Total RNA Nano Assay (Agilent Technologies, Santa Clara, CA, United States). sRNAs shorter than 30 nt were isolated from the gel following acrylamide gel electrophoresis. Libraries for sequencing were prepared using the TruSeq small RNA kit (Illumina, San Diego, CA, United States). Sequencing was carried out with an Illumina HiSeq 2500 instrument using the 50-bp single-end mode. RNA sequences (i.e., reads) were sorted to files based on their size after base-calling and trimming of the adapter sequences and were subjected to further analysis. Data were deposited to the European nucleotide archive (ENA), Accession No. ERP108051^2^.

Detection sensitivity achieved by sRNA deep sequencing as compared with RT-RT-PCR was compared using an RT-RT-PCR -test commercially available from Fera Science, Ltd. (York, United Kingdom) (Boonham et al., 2008) in the plant analysis laboratory unit of the Finnish Food Safety Authority Evira. Because the PCR primers in Fera’s method are designed for detection of PVY or PVA, it was not possible to distinguish the three infectious PVA clones (PVA-GFP, PVA-RFP, and PVA-GUS) included in the pools. Therefore, RNA extracted from leaves of the tobacco plants, each infected with one of the three PVA constructs, was separately diluted to four different concentrations (**Table 1**). For PVY, aliquots of the original pools sent for sequencing were tested by RT-RT-PCR (**Table 1**). All RNA samples hence prepared were transferred to test tubes containing 3 μg of a mixture of total RNA extracted from leaves of strawberry, raspberry and *Ribes* spp. in a total volume of 15 μl. This was done to mimic the total RNA concentrations used in sRNA sequencing. All the thus generated 16 samples were tested by RT-RT-PCR in duplicate using 50 ng of RNA. The experiment was carried out three times starting from the dilution of RNA.

### Bioinformatics

Velvet software was used to produce contiguous sequences (contigs) from the reads of 21–24 nt (Zerbino and Birney, 2008). The k-mer values 13, 15, and 17 were tested for assembly of contigs. The length cut-off values (COVs) that determined whether the contigs would be analyzed further were 30, 50, and 100 nt. Subsequently, contigs assembled using k-mer values 13–25 were post-assembled into larger contigs using AssemblyAssembler^3^.

Homologous sequences were sought in the National Center for Biotechnology Information (NCBI) database using BLASTn (nucleotide blast). The contigs corresponding to the reference sequences were identified by alignment with the GFP-encoding (741 nt; NCBI Accession No. 13928062), RFP-encoding (678 nt; Accession No. 125976381) or GUS-encoding (1812 nt; Accession No. S69414.1) sequence and the sequence of PVY (9701 nt; Accession No. X97895.1) using BLASTn.

The mapping analysis of sequence data was carried out with Novoalign software (Novocraft Technologies, Malaysia). All obtained 21- to 24-nt reads were analyzed against the reference sequences.

## Results

The amounts of RNA from PVY and the three engineered clones of PVA, PVA-GFP, PVA-RFP, and PVA-GUS, in the uppermost fully expanded leaves of the infected tobacco plants were 15.08, 0.86, 2.20, and 11.12 ng per 1 μg of total RNA, respectively, as determined by RT-qPCR. Based on this knowledge, different amounts of RNA were added to the RNA pools (**Table 1**) and subjected to deep sequencing.

Analysis of the RNA pools with the Bioanalyzer assay at Fasteris SA indicated that the quality of the RNA was good enough for sRNA sequencing [RNA integrity number (RIN) values 4.6–5.8]. RNA concentrations in the pools measured with Qubit at Fasteris SA (**Table 2**) differed from the expected concentration of 200 ng/μl, which was originally based on the Nanodrop measurements. Sequencing by Illumina was successful, as indicated by a mean quality score > 38 in each pool. Over 97% of the reads had quality scores > 30 as determined by Illumina. The total number of reads per pool was 14–21 million (**Table 2**). Most reads were 21–24 nt (**Table 3**), but reads of up to 44 nt were also detected as a result of imperfect size selection by acrylamide gel electrophoresis and subsequent purification of the sRNAs.

**Table 2:** Concentration of RNA in pools of samples measured by Qubit, and the total number of reads obtained by sRNA deep sequencing.

Pool^a^	RNA concentration (ng/μl)	Total reads (≤44 nt)
1	188	19,363,413
2	209	21,076,337
3	176	17,745,584
4	172	14,352,860


^*a*^Pools correspond to those shown in **Table 1**.

**Table 3:** Number of contigs assembled from sRNAs by Velvet (k-mer = 15, COV = 50 nt) and identified as the references sequences by BLASTn in four pools of samples.

Pool^a^	Amount of reference RNA added (ng)^b^	Reference sequence and length (nt)^b^	Contigs specific to reference sequences (BLASTn)		Total no. of sequenced sRNAs (21–24 nt)	Total no. and length of contigs
			Number	Length (nt)		
1	1	PVY (9701)	46	≤926	14,159,712	2013 (≤926)
	1	GUS (1812)	13	≤237		
	0.01	RFP (678)	4	≤110		
	0.00002	GFP (741)	0	–		
2	0.2	RFP (678)	3	≤513	15,856,030	3081 (≤652)
	0.01	PVY (9701)	54	≤220		
	0.0002	GFP (741)	0	–		
	0.00001	GUS (1812)	0	–		
3	0.1	GFP (741)	6	≤235	12,332,818	3255 (≤739)
	0.002	GUS (1812)	5	≤102		
	0.0001	PVY (9701)	0	–		
	0.000001	RFP (678)	0	–		
4	0.02	GUS (1812)	15	≤196	9,856,044	3218 (≤391)
	0.0001	RFP (678)	0	–		
	0.000002	GFP (741)	0	–		
	0.000001	PVY (9701)	0	–		


*^a^Pools correspond to those shown in **Table 1**.*

*^b^Amounts of PVY and PVA RNA added to a mixture of total RNA from leaves of strawberry, raspberry and *Ribes* spp. PVA was engineered to carry a reference gene encoding beta-glucuronidase (GUS) or green (GFP) or red (RFP) fluorescent protein. Lengths of the reference RNAs (in nucleotides) are shown in parentheses.*

Contigs were built with Velvet using 21- to 24-nt reads. The k-mer values 13, 15, and 17 and COVs at 30, 50, and 100 nt were tested. The highest numbers of contigs homologous to PVY or to the reference sequences inserted into the genome of PVA were obtained using the k-mer value 15 and a COV of 50 nt (**Table 3**). Using a COV of 100 nt led to the loss of shorter contigs specific to the reference sequences, whereas a COV of 30 nt produced short contigs that were not homologous to the reference sequences (data not shown).

PVY was detected by Velvet-based *de novo* analysis in the RNA pools amended with total RNA of tobacco containing 1 or 0.01 ng of PVY RNA (**Table 3**). However, no PVY-specific contigs were obtained from the pools containing 0.001 or 0.000001 ng PVY RNA.

*Potato virus A* genomes, each carrying a different reference sequence encoding GUS, GFP or RFP, were distinguished by the reference sequence-specific contigs obtained by Velvet-based *de novo* analysis of sRNA reads. GUS-specific contigs were obtained from the pools containing 1, 0.02, or 0.002 ng of PVA-GUS RNA. GFP was detected in the pool containing 0.1 ng PVA-GFP RNA, but not in the three other pools containing 500–50,000 times less PVA-GFP RNA. RFP-specific contigs were obtained from pools containing 0.2 and 0.01 ng of PVA-RFP RNA (**Table 3**).

Post-assembly analysis of the data with AssemblyAssembler was carried out using a wider range of k-mers (13–25 nt). Results were essentially similar to those obtained with Velvet alone, but contigs were longer in some cases when AssemblyAssembler was used.

**FIGURE 1:**
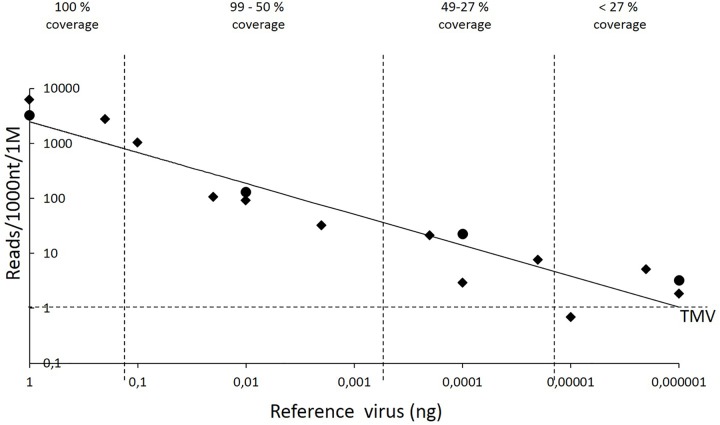
Reference sequence–guided analysis of the obtained 21- to 24-nt reads. The descending trend line (*y* = 2486.1 × 0.5616; correlation coefficient, 0.8927) illustrates the correlation between the number of aligned reads and the amount of viral RNA. Novoalign software was used to align the sRNA reads to the reference sequences. The *y* axis shows the number of reads that aligned per 1000 nt of the reference sequence per 1 million (1 M) total reads, i.e., genomic sequence of PVY or the marker gene sequences GFP, GUS, and RFP, each carried by a different copy of the PVA genome. The *x* axis indicates the amount of PVY or PVA genomic RNA. The sequenced RNA pool of 15 μl also contained 3 μg of total RNA obtained by mixing RNA from strawberry, raspberry, and *Ribes* spp. Quadrilaterals refer to PVY, whereas circles refer to the three reference sequences carried by PVA. Both axes are in a logarithmic scale. The vertical dashed lines were included to visualize how diminishing amounts of viral RNA reduce the coverage of reference sequences by sRNA reads and hence limit the possibility of detecting viruses. The horizontal dashed line indicates the background noise caused by alignment of a few reads to the sequence of *Tobacco mosaic virus* (TMV), which was not included in the samples.

All sequenced 21- to 24-nt reads were aligned with the reference sequences using Novoalign to determine the relationship between the amount of virus and the number of reads aligned to the reference sequences. Because the reference sequences varied in length and the total number of reads varied between the sample pools, the results were normalized among the four pools by calculating the number of reads aligned per 1000 nt of the reference sequence and per 1 million total reads. The number of reads that aligned to the reference sequences decreased with diminishing amounts of viral RNA (**Figure 1**).

Aligning the 21- to 24-nt reads from each pool to the genome of *Tobacco mosaic virus* (TMV; NCBI Accession No. JX993906.1 – not present in the samples) revealed that, on average, only one read was perfectly aligned per 1000 nt of the TMV genome per 1 million total reads. Similar results were observed in all pools, indicating that only a low level of background noise was present in the analysis.

Detection of the reference sequences by the Velvet-based *de novo* analysis required 0.002 ng of viral RNA (pool 3, GUS-encoding sequence, **Table 3**). Novoalign-based mapping of all the 21- to 24-nt reads from pool 3 to the *GUS* sequence reached nearly complete coverage (99%), with the mean depth of coverage being 12, i.e., each nucleotide in the reference sequence was covered by 12 reads on average. Smaller amounts of reference sequence-carrying viral RNA (0.00002–0.0002 ng) resulted in coverage of ca.25–50% of the reference sequence, whereas lower amounts of viral RNA (≤0.00001 ng) resulted in only sporadic read alignments to the corresponding reference sequence; however, the depth of coverage in those few positions was high in some cases.

The standard RT-RT-PCR method (“Fera’s method”) used in the plant analysis laboratory unit of the Finnish Food Safety Authority Evira detected PVA and PVY in the pools containing ≥0.0002 ng of PVA RNA and ≥0.0001 ng of PVY RNA in 3 μg of total RNA, combined from miscellaneous plants, in a volume of 15 μl. The average Cq-values were 34.63 and 34.59 for the pools containing 0.0002 ng of PVA and 0.0001 ng of PVY RNA, respectively, which corresponds to 3.0 and 1.5 fg of viral RNA, respectively, in 50 ng of total RNA used for an RT-RT-PCR reaction. No amplification products were obtained when the RNA pools were tested by RT-RT-PCR without adding any PVY or PVA RNA (data not shown).

## Discussion

There is increased interest in applying sRNA-based approaches to virus detection, as well as to official plant inspection. The aim of this study was to estimate sensitivity of the sRNA-based method, as compared with the established RT-RT-PCR approach. The question was addressed using two globally distributed, aphid-transmitted potyviruses, PVY and PVA, in the experiments. Three marker genes inserted in an infectious cDNA clone of PVA allowed us to distinguish the three engineered PVA clones and treat them as ‘strains’ of PVA. Total RNA from various crop plants was included in the samples to be analyzed by sRNA and RT-RT-PCR, to maintain the overall amount and concentration of RNA as constant as possible across the samples.

In this study, sRNA libraries were prepared from gel-extracted small RNA fractions (<30 nt) obtained from 3 μg of plant total RNA, resulting in 15–20 million reads per sample. Results showed that detection of PVA and PVY by the specified sRNA approach using Velvet-based *de novo* analysis required a 10-fold higher amount of viral RNA than detection by RT-RT-PCR. sRNA deep sequencing followed by Velvet-based *de novo* assembly of the reads to contigs ≥ 50 nt required 0.002 ng of viral RNA in 3 μg of total RNA in a volume of 15 μl. The *de novo* assembly approach is needed when no educated presumption is available about the viruses present. This is an advantage of sRNA-based virus detection, because sRNA-based diagnosis using *de novo* assembly of the reads can detect all viruses without the need for virus- or virus group-specific primers or probes. Higher amounts of viral sRNA allowed assembly of longer contigs and more reliable virus identification by comparison to the sequences available in databases such as NCBI.

In contrast, sRNA-based virus detection was 10 times more sensitive than RT-RT-PCR when knowledge was available about the viruses likely present in the samples. In this case, sRNA reads were mapped to the existing reference sequences suspected in the samples using the Novoalign software. Hence, as little as 0.00002–0.0002 ng of PVY or PVA RNA was sufficient to cover ca. 25–50% of the reference sequence with virus-derived sRNA reads. This coverage, albeit not complete, was high enough to conclude the presence of PVA and PVY. Higher amounts of viral RNA resulted in nearly full to complete coverage of the reference sequences. The RT-RT-PCR method used in the plant analysis laboratory unit of the Finnish Food Safety Authority Evira detected PVA and PVY in the pools containing ≥0.0002 and ≥0.0001 ng of PVY and PVA RNA, respectively. Hence, in this case sRNA-based diagnosis reached a similarly high sensitivity relative to RT-RT-PCR in the detection of the viruses.

Taken together, deep sequencing using the specific conditions described in this study and subsequent *de novo* analysis required the equivalent of 30 fg of viral RNA in 50 ng of total RNA per PCR reaction for detection of the viruses. However, with prior knowledge about the possible viruses present in the samples, detection sensitivity reached the level of 0.3 fg of viral RNA in 50 ng of total RNA per PCR reaction. The detection limit for PVY and PVA by RT-qPCR in this study was 3 and 1.5 fg of viral RNA, respectively, in 50 ng of total RNA per PCR reaction.

It is worth noting that the sensitivity of sRNA deep sequencing for virus detection can be adjusted during the process of sample preparation for sequencing and by depth of sequencing. For example, starting the library preparation from total RNA without gel-based or column-based separation of the sRNA fraction could diminish the sensitivity, because unwanted RNA remains in the library. Size selection can also be conducted using ready-made libraries. Deeper sequencing, i.e., obtaining more reads per sample, is expected to increase the sensitivity of virus detection but will also increase the cost of the analysis.

Recent studies have compared sRNA-based virus diagnostics with sequencing of long viral RNAs (RNAseq) as two alternative approaches for virus detection based on next-generation sequencing. Both methods are also able to detect previously unknown viruses (Pecman et al., 2017; Zheng et al., 2017; and references therein). Those authors concluded that all plant viruses are detectable using both methods. The costs of sRNA-based diagnosis may be slightly lower, but more user-friendly bioinformatics tools designed for the purpose of sRNA data analysis would be appreciated for routine use of the method in diagnostic labs that must analyze vast numbers of samples in a limited time.

## Author Contributions

JS made experimental design and carried out laboratory experiments. JS and JV interpreted the data and wrote the manuscript.

## Conflict of Interest Statement

The authors declare that the research was conducted in the absence of any commercial or financial relationships that could be construed as a potential conflict of interest.

## Footnotes

1 https://academic.oup.com/nar/article/40/15/e115/1223759

2 www.ebi.uk/ena

3 https://github.com/dzerbino/velvet/tree/master/contrib/AssemblyAssembler1.3
